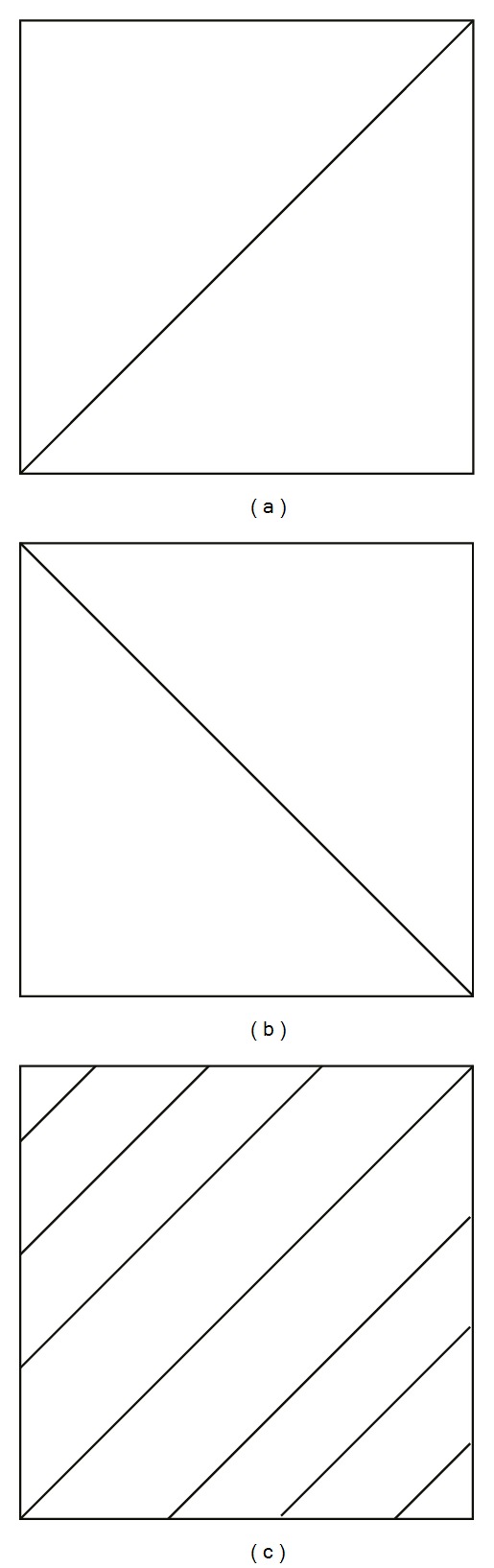# Characterizations of MV-Algebras Based on the Theory of Falling Shadows

**DOI:** 10.1155/2014/951796

**Published:** 2014-08-28

**Authors:** Yongwei Yang, Xiaolong Xin, Pengfei He

**Affiliations:** ^1^School of Mathematics and Statistics, Anyang Normal University, Anyang 455000, China; ^2^School of Mathematics, Northwest University, Xi'an 710127, China

## Abstract

Based on the falling shadow theory, the concept of falling fuzzy (implicative) ideals as a generalization of that of a *T*_∧_-fuzzy (implicative) ideal is proposed in MV-algebras. The relationships between falling fuzzy (implicative) ideals and *T*-fuzzy (implicative) ideals are discussed, and conditions for a falling fuzzy (implicative) ideal to be a *T*_∧_-fuzzy (implicative) ideal are provided. Some characterizations of falling fuzzy (implicative) ideals are presented by studying proprieties of them. The product *⊛* and the up product *⊚* operations on falling shadows and the upset of a falling shadow are established, by which *T*-fuzzy ideals are investigated based on probability spaces.

## 1. Introduction

Nonclassical logics take advantage of classical logics to handle information and uncertainty, and they become a formal and useful tool for dealing with fuzzy information and uncertain information in computer science. MV-algebras as the algebraic counterpart of many-valued prepositional calculus were proposed by Chang [1]. The classical two-valued logic gives rise to the study of Boolean algebras and every Boolean algebra will be an MV-algebra whereas the converse does not hold. The prototypical model of MV-algebras is based on the real interval [0,1]. Motivated by the search for adequate algebraic structure for the quantum counterpart of the real interval [0,1], Giuntini [2] introduced the notion of QMV-algebras which is a nonlattice theoretic generalization of MV-algebras. From an algebraic point of view, MV-algebras and QMV-algebras share a common set of axioms, which was called supplement algebras (S-algebras). What makes an S-algebra an MV-algebra is the addition of the Łukasiewicz axiom, and it also makes MV-algebra a lattice ordered structure. MV-algebras form a category which is equivalent to the category of abelian lattice ordered groups (*l*-groups, for short) with strong units [3]. These make the interest in MV-algebras relevant outside the realm of logic.

The ideal theory plays an important role in studying logical algebras. From the logic point of view, the sets of provable formulas in corresponding systems can be described by (fuzzy) ideals of those algebraic semantics. Some types of ideals in MV-algebras have been widely studied and many important results are obtained [4, 5]. Belluce and Di Nola [6] gave the definition of Łukasiewicz rings and derived that MV-algebras arising as the MV-algebra of ideals of a commutative ring are exactly the complete and atomic ones. Lele and Nganou [7] introduced the notion of ideals in BL-algebras as a natural generalization of that of ideals in MV-algebras and proved that quotient BL-algebras turn out to be MV-algebras. It is also proved that fuzzy Boolean ideals coincide with fuzzy implicative ideals in MV-algebras [8]. Jun and Walendziak [9] applied the fuzzy set theory to ideals of pseudo MV-algebras and introduced the notion of fuzzy (implicative) ideals; moreover, [10] extended the notions of fuzzy ideals to (∈, ∈∨ *q*)-fuzzy (implicative) ideals by using the concept of quasicoincidence of a fuzzy value with a fuzzy set.

Falling shadow representation theory was introduced by Goodman [11] and Wang and Sanchez [12] independently, and it directly relates probability concepts to the membership function of fuzzy sets, just as Goodman pointed out that the equivalence of a fuzzy set and a class of random sets aims to study a unified treatment of uncertainty modelled by means of combining probability and fuzzy set theory. Tan et al. [13, 14] established a theoretical approach for defining a fuzzy inference relation and fuzzy set operations based on the theory of falling shadows. Yuan and Lee [15] gave a theoretical approach of the fuzzy algebraic system based on the mathematical structure of the falling shadow theory which was formulated in [16]. The characterization of the approach is that a fuzzy subalgebraic system is considered as the falling shadow of the cloud of the subalgebraic system. The falling shadow theory was also applied to study subalgebras and ideals of BCK/BCI-algebras [17, 18] and *d*-algebras [19]. Inspired by [15], Yu et al. investigated falling fuzzy ideals of a hemiring [20] and falling fuzzy filters of a BL-algebra [21] based on the theory of falling shadows and fuzzy sets, which provide a theoretical approach for the further studying of fuzzy ideals in MV-algebras.

The paper aims to investigate ideals of MV-algebras based on the falling shadow theory. The notion of falling fuzzy (implicative) ideals is introduced, and then some properties of them are studied. It is pointed out that a falling fuzzy (implicative) ideal is a *T*_∧_-fuzzy (implicative) ideal and some conditions under which a falling fuzzy (implicative) ideal becomes a *T*_∧_-fuzzy (implicative) ideal are provided. We also derive several characterizations of falling fuzzy (implicative) ideals. *T*-fuzzy ideals are investigated based on the probability space by introducing the product and up product operations and the upset of a falling shadow.

## 2. Preliminaries

In the section, we present some definitions and results about MV-algebras for purpose of reference.

**Definition 1 (see [22]).**

A *t*-norm is a binary operation *T* on [0,1]  (i.e., *T* : [0,1]^2^ → [0,1]) satisfying the following conditions:

(i) *T*  is commutative and associative; that is, for any *x*, *y*, *z* ∈ [0,1],
  (1) $$\begin{array}{c} T\left(x,y\right)=T\left(y,x\right),\\ T\left(T\left(x,y\right),z\right)=T\left(x,T\left(y,z\right)\right); \end{array}$$
(ii) *T* is nondecreasing in both arguments; that is,
  (2) $$\begin{array}{c} x_{1}\leq x_{2}\;\;\text{implies}\;\;T\left(x_{1},y\right)\leq T\left(x_{2},y\right),\\ y_{1}\leq y_{2}\;\;\text{implies}\;\;T\left(x,y_{1}\right)\leq T\left(x,y_{2}\right); \end{array}$$
(iii) *T*(1, *x*) = *x* and *T*(0, *x*) = 0 for any *x* ∈ [0,1].

*T* is a continuous *t*-norm if it is a *t*-norm and is a continuous mapping of [0,1]^2^ into [0,1].

**Example 2 (see [22]).**

The following are some important examples of continuous *t*-norms:

1. Łukasiewicz *t*-norm *T*_*m*_(*x*, *y*) = max⁡{0, *x* + *y* − 1};
2. Gödel *t*-norm: *T*_∧_(*x*, *y*) = min⁡{*x*, *y*};
3. product *t*-norm: *T*_*p*_(*x*, *y*) = *x* · *y*.

**Lemma 3 (see [22]).**

Let *T* be a continuous *t*-norm. Then there is a unique operation *x* → *y* satisfying the condition *T*(*x*, *z*) ≤ *y* if and only if *z* ≤ *x* → *y* for all *x*, *y*, *z* ∈ [0,1]; namely, *x* → *y* = max⁡{*z*∣*T*(*x*, *z*) ≤ *y*}.

The operation *x* → *y* from Lemma 3 is called the residuum of the *t*-norm.

**Definition 4 (see [1, 23]).**

An MV-algebra is an algebra (*A*, ⊕, ¬, 0) of type (2,2, 0) satisfying the following equations: for any *x*, *y*, *z* ∈ *A*,

(MV1) *x* ⊕ (*y* ⊕ *z*) = (*x* ⊕ *y*) ⊕ *z*;
(MV2) *x* ⊕ *y* = *y* ⊕ *x*;
(MV3) *x* ⊕ 0 = *x*;
(MV4) ¬¬*x* = *x*;
(MV5) *x* ⊕ ¬0 = ¬0;
(MV6) ¬(¬*x* ⊕ *y*) ⊕ *y* = ¬(¬*y* ⊕ *x*) ⊕ *x*.

Let (*A*, ⊕, ¬, 0) be an MV-algebra. For any *x*, *y* ∈ *A*, we define 1 = ¬0, *x* ⊗ *y* = ¬(¬*x* ⊕ ¬*y*), *x* → *y* = ¬*x* ⊕ *y*, *x* ⊖ *y* = *x* ⊗ ¬*y*, *x*∨*y* = ¬(¬*x* ⊕ *y*) ⊕ *y* = (*x* ⊖ *y*) ⊕ *y*, *x*∧*y* = ¬(¬*x*∨¬*y*), 0*x* = 0, (*n* + 1)*x* = *nx* ⊕ *x*, *x* ^0^ = 1, and *x* ^(*n*+1)^ = *x* ^*n*^ ⊗ *x* for any *n* ≥ 0; then (*A*, ∧, ∨, 0,1) is a bounded distributive lattice [23].

From now on, (*A*, ⊕, ¬, 0) is an MV-algebra unless otherwise mentioned, which will often be referred to by its support set *A*. Here we summarize the necessary notions and some previous results which will be used in the sequel.

**Lemma 5 (see [23, 24]).**

In any MV-algebra *A*, the following properties hold: for any *x*, *y*, *z* ∈ *A*,

1. *x* ≤ *y* if and only if ¬*x* ⊕ *y* = 1 if and only if *x* ⊖ *y* = 0;
2. *x* ⊗ ¬*x* = 0, *x* ⊕ ¬*x* = 1, *x* ⊗ *y* = *y* ⊗ *x*;
3. *x* ⊗ *y* ≤ *x*∧*y* ≤ *x*∨*y* ≤ *x* ⊕ *y*;
4. (*x* ⊖ *y*) ⊖ *z* = (*x* ⊖ *z*) ⊖ *y* = *x* ⊖ (*y* ⊕ *z*);
5. *x*∧*y* = *x* ⊖ (*x* ⊖ *y*) = *y* ⊖ (*y* ⊖ *x*);
6. *x* ≤ *y* implies ¬*y* ≤ ¬*x*, *x* ⊗ *z* ≤ *y* ⊗ *z*, and *x* ⊕ *z* ≤ *y* ⊕ *z*;
7. *x* ⊗ (*y*∨*z*) = (*x* ⊗ *y*)∨(*x* ⊗ *z*), *x* ⊕ (*y*∨*z*) = (*x* ⊕ *y*)∨(*x* ⊕ *z*);
8. *x* ⊖ *y* ≤ (*x* ⊖ *z*)⊕(*z* ⊖ *y*), (*x* ⊖ *z*)⊖(*y* ⊖ *z*) ≤ *x* ⊖ *y*.

If a mapping over a set *X* is defined as follows: for any *x* ∈ *X*,

(3) $$\begin{array}{c} \mu :X⟶\left[0,1\right],\;x⟼\mu \left(x\right), \end{array}$$

then *μ* is called a fuzzy set over *X*, where *μ*(*x*) is the degree of membership of *x* with respect to *μ* [25].

Letting *X* be a nonempty set, denote the set of all fuzzy sets of *X* by *F*(*X*). For any *μ*, *ν* ∈ *F*(*X*), define *μ*⊆*ν*: *μ*(*x*) ≤ *ν*(*x*) for any *x* ∈ *X*. *μ* = *ν* implies that *μ*⊆*ν* and *ν*⊆*μ* hold. The fuzzy sets *μ*⊔*ν* and *μ*⊓*ν* over *X* are defined by (*μ*⊔*ν*)(*x*) = *μ*(*x*)∨*ν*(*x*) = max⁡{*μ*(*x*), *ν*(*x*)}, (*μ*⊓*ν*)(*x*) = *μ*(*x*)∧*ν*(*x*) = min⁡{*μ*(*x*), *ν*(*x*)}, for any *x* ∈ *X*.

The theory of falling shadows is an important tool in the theoretical developments and practical applications of fuzzy sets, and some of their properties and notions are displayed in the following. For further information, the readers can be referred to [16, 26].

Given a universal set *U*, let *P*(*U*) denote the power set of *U*. For any *u* ∈ *U*, let $\dot{u}=\{E|u\in E,E\subseteq U\}$ and for any *E*⊆*U* let $\dot{E}=\{\dot{u}|u\in E\}$.

An order pair (*P*(*U*), *B*) is called to be a hypermeasurable structure on *U* if *B* is a *σ*-field in *P*(*U*) and $\dot{U}\subseteq B$.

Let (*Ω*, *A*, *P*) be a probability space and (*P*(*U*), *B*) a hypermeasurable structure on *U*. If a mapping *ξ* : *Ω* → *P*(*U*) is *A* → *B* measurable, that is, ∀*C* ∈ *B*,

(4) $$\begin{array}{c} \xi^{-1}\left(C\right)=\left\{\omega \in \Omega \;|\;\xi \left(\omega \right)\in C\right\}\in A, \end{array}$$

then *ξ* is called a random set on *U*.

**Figure 1:**
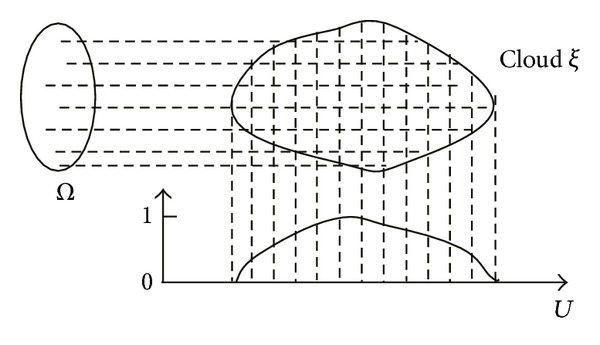
Falling shadow $\tilde{H}$.

Let *ξ* be a random set on *U*. For any *u* ∈ *U*, if $\tilde{H}(u):=P\{\omega |u\in \xi (\omega )\}$, then $\tilde{H}$ is a fuzzy set of *U*. The fuzzy set $\tilde{H}$ is called a falling shadow of the random set *ξ*, and *ξ* is called a cloud of $\tilde{H}$ (see Figure 1).

Let (*Ω*, *A*, *P*) be a probability space, where *P* is a probability distribution of two-dimensional random variables (*ξ*, *η*) on [0,1]^2^. There are many types of probability distributions, but only three types are the most classic ones (see Figure 2).

1. If the whole probability *P* is concentrated and uniformly distributed on the main diagonal of the unit square [0,1]^2^, then *P* is called a diagonal distribution.
2. If the whole probability *P* is concentrated and uniformly distributed on the antidiagonal of the unit square [0,1]^2^, then *P* is called an antidiagonal distribution.
3. If the whole probability *P* is uniformly distributed on the unit square [0,1]^2^, then *P* is called an independent distribution.

A nonempty set *I* of *A* is called an ideal of *A* if it satisfies the following conditions: ∀*x*, *y* ∈ *A*, (1)  *x*, *y* ∈ *I* implies *x* ⊕ *y* ∈ *I*; (2)  *x* ≤ *y* and *y* ∈ *I* imply *x* ∈ *I*. For purpose of convenience, let *∅* be an ideal of *A* in the the rest of the sections. If an ideal *I* satisfies the following condition: (*x* ⊖ *y*) ⊖ *z* ∈ *I* and *y* ⊖ *z* ∈ *I* imply *x* ⊖ *z* ∈ *I* for any *x*, *y*, *z* ∈ *A*, then *I* is called an implicative ideal of *A*.

**Proposition 6 (see [1]).**

Let *I* be a nonempty set of *A*. Then the following statements are equivalent:

1. *I* is an ideal of *A*;
2. 0 ∈ *I*; ∀*x*, *y* ∈ *A*, *x* ⊖ *y* ∈ *I*, and *y* ∈ *I* imply *x* ∈ *I*;
3. ∀*x*, *y*, *z* ∈ *A*, if *z* ⊖ *x* ≤ *y* and *x*, *y* ∈ *I*, then *z* ∈ *I*.

## 3. Falling Fuzzy Ideals

In this section, we will introduce the notions of *T*-fuzzy ideals and falling fuzzy ideals of MV-algebras. The relationships between *T*-fuzzy ideals and falling fuzzy ideals are provided, and some characterizations of falling fuzzy ideals are displayed.

**Definition 7 .**

A fuzzy set *μ* of *A* is called a *T*-fuzzy ideal of *A*, if *μ* satisfies ∀*x*, *y* ∈ *A*,

1. *μ*(*x* ⊕ *y*) ≥ *T*(*μ*(*x*), *μ*(*y*));
2. *x* ≤ *y* implies *μ*(*x*) ≥ *μ*(*y*).

It is easy to see that a *T*-fuzzy ideal of *A* is a *T*_∧_-fuzzy ideal when *T* = *T*_∧_ and a *T*_∧_-fuzzy ideal is also called a fuzzy ideal [4]. We denote the set of all *T*-fuzzy ideals of *A* by TFI(*A*). The next result can be proved similar to that for *T*_∧_-fuzzy ideals of *A*.

**Theorem 8 .**

Let *μ* be a fuzzy set of *A*. Then *μ* is a *T*-fuzzy ideal of *A* if and only if it satisfies the following conditions: ∀*x*, *y* ∈ *A*,

1. *μ*(0) ≥ *μ*(*x*);
2. *μ*(*x*) ≥ *T*(*μ*(*y*), *μ*(*x* ⊖ *y*)).

**Proposition 9 .**

Let *μ* be a fuzzy set of *A*. Then the following conditions are equivalent:

1. *μ* is a *T*_∧_-fuzzy ideal of *A*;
2. ∀*x* ∈ *A*, *μ*(0) ≥ *μ*(*x*); ∀*x*, *y* ∈ *A*, *μ*(*x*) ≥ min⁡{*μ*(*y*), *μ*(*x* ⊖ *y*)};
3. ∀*t* ∈ [0,1], *L*(*μ*; *t*) is an ideal of *A*, where *L*(*μ*; *t*) = {*x* ∈ *A*∣*μ*(*x*) ≥ *t*}.

In the following, we give some properties of *T*-fuzzy ideals for the further discussion.

**Proposition 10 .**

Let *μ*_1_, *μ*_2_ be fuzzy sets of *A*. If *μ*_1_, *μ*_2_ are *T*-fuzzy ideals of *A*, then *μ*_1_⊓*μ*_2_ is a *T*-fuzzy ideal of *A*.

**Proof**

The proof is straightforward.

As a direct consequence of Proposition 10, we have the following result.

**Corollary 11 .**

Let *μ*_*i*_  (*i* ∈ Λ) be fuzzy sets of *A*, where Λ is an index set. If *μ*_*i*_ is a *T*-fuzzy ideal of *A*, then ⊓_*i*∈Λ_ *μ*_*i*_ is a *T*-fuzzy ideal of *A*.

**Example 12 .**

Let *A* = {0, *a*, *b*, 1} where 0 < *a* < 1, 0 < *b* < 1. Define the operations ⊕ and ¬ on *A* as follows:

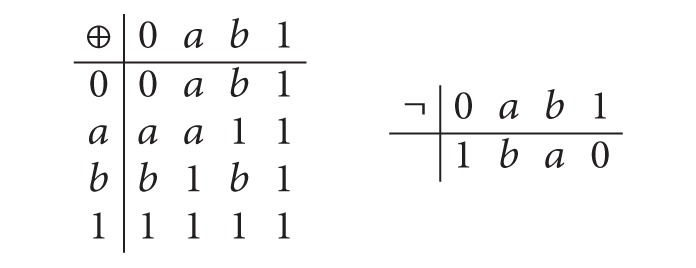
(5)

It is clear that (*A*, ⊕, ¬, 0) is an MV-algebra. Define fuzzy sets *μ*_1_ and *μ*_2_ of *A* as follows:

(6) $$\begin{array}{c} \mu_{1}\left(x\right)=\{\begin{array}{cc} 0.9, & x=0,\\ 0.6, & x=a,\\ 0.3, & x=b,1; \end{array}\;\;\mu_{2}\left(x\right)=\{\begin{array}{cc} 0.7, & x=0,\\ 0.5, & x=b,\\ 0.2, & x=a,1. \end{array} \end{array}$$

Then *μ*_1_ and *μ*_2_ are *T*_∧_ fuzzy ideals of *A*, but *μ*_1_⊔*μ*_2_ is not a *T*_∧_ fuzzy ideal, since (*μ*_1_⊔*μ*_2_)(*a* ⊕ *b*) = 0.3 < (*μ*_1_⊔*μ*_2_)(*a*)∧(*μ*_1_⊔*μ*_2_)(*b*) = 0.6∧0.5 = 0.5.

However, the union of two *T*-fuzzy ideals is not a *T*-fuzzy ideal. In order to investigate the algebraic properties of the set of all *T*_∧_-fuzzy ideals in BL-algebras, [27, 28] introduced the notion of generated fuzzy ideals in BL-algebras. At first we borrow the notion and modify it for our reasons.

**Definition 13 .**

Let *μ* be a fuzzy subset of *A*. A *T*-fuzzy ideal *ν* of *A* is said to be generated by *μ*, if *μ* ≤ *ν* and *μ* ≤ *λ* implies  *ν* ≤ *λ* for any *T*-fuzzy ideal *λ* of *A*. The fuzzy ideal generated by *μ* will be denoted by (*μ*]_*T*_.

The *T*-fuzzy ideal of *A* generated by a fuzzy subset *μ* is the least *T*-fuzzy ideal of *A* containing *μ*. It is also the intersection of all the *T*-fuzzy ideals of *A* containing *μ*.

The following theorem shows how to construct the *T*-fuzzy ideal generated by a fuzzy subset.

**Theorem 14 .**

Let *μ* be a fuzzy subset of *A*. If the fuzzy subset μ* of *A* is defined by μ*(*a*) = sup⁡{*T*(⋯*T*(*T*(*μ*(*x*_1_), *μ*(*x*_2_)), *μ*(*x*_2_)),…, *μ*(*x*_*n*_))∣*a* ≤ *x*_1_ ⊕ *x*_2_ ⊕ ⋯⊕*x*_*n*_; *x*_1_, *x*_2_,…, *x*_*n*_ ∈ *A*} for any *a* ∈ *A*, then μ* = (*μ*]_*T*_.

For any *μ*_1_, *μ*_2_∈ TFI(*A*), we define *μ*_1_⨄*μ*_2_ = (*μ*_1_⊔*μ*_2_]_*T*_. Generally, for any *μ*_*i*_∈ TFI(*A*)  (*i* ∈ Λ), ⨄_*i*∈Λ_ *μ*_*i*_ : = (⊔_*i*∈Λ_ *μ*_*i*_]_*T*_. Similar to Theorem  38 in [28], we can obtain the following result.

**Theorem 15 .**

(TFI(*A*), ⊓, ⨄, 0_*A*_, 1_*A*_) is a complete modular lattice with 0_*A*_ and 1_*A*_ as the least lower bound and the largest upper bound of TFI(*A*), respectively, where 0_*A*_(*x*) = 0 and 1_*A*_(*x*) = 1 for any *x* ∈ *A*.

In what follows, we will introduce the notion of falling fuzzy ideals and then investigate their properties.

**Definition 16 .**

Let (*Ω*, *A*, *P*) be a probability space and let *ξ* : *Ω* → *P*(*A*) be a random set. If *ξ*(*ω*) is an ideal of *A* for any *ω* ∈ *Ω*, then the falling shadow $\tilde{H}$ of the random set *ξ*, that is, ∀*u* ∈ *A*,

(7) $$\begin{array}{c} \tilde{H}\left(u\right)=P\left(\omega \;|\;u\in \xi \left(\omega \right)\right), \end{array}$$

is called a falling fuzzy ideal of *A*.

For better understanding the definition of falling fuzzy ideals, we illustrate it by the following example.

**Example 17 .**

Let *A* = {(1, *y*) ∈ *R* ^2^∣*y* ≥ 0}∪{(2, *y*) ∈ *R* ^2^∣*y* ≤ 0}, 0 = (1,0), 1 = (2,0). For any (*a*, *b*), (*c*, *d*) ∈ *A*, we define operations ⊕ and ¬ as follows:

(8) $$\begin{array}{c} \left(a,b\right)⊕\left(c,d\right)=\{\begin{array}{cc} \left(1,b+d\right), & \text{if}\;\;a=c=1,\\ \left(2,ad+b\right), & \text{if}\;\;ac=2\;\;\text{and}\;\;ad+b\leq 0,\\ \left(2,0\right), & \text{in}\;\;\text{other}\;\;\text{cases}, \end{array}\\ \neg \left(a,b\right)=\left(\frac{2}{a},-\frac{2b}{a}\right). \end{array}$$

It is easy to verify that (*A*, ⊕, ¬, 0) is an MV-algebra.

Let (*Ω*, *A*, *P*) = ([0,1], *A*, *m*), where *A* is a Borel field on [0,1] and *m* is the usual Lebesgue measure. Denote *A*_1_ = {(1, *y*) ∈ *R* ^2^∣*y* > 0} and *A*_2_ = {(2, *y*) ∈ *R* ^2^∣*y* < 0}. The mapping *ξ* : *Ω* → *P*(*A*) is defined by

(9) $$\begin{array}{c} \xi \left(t\right)=\{\begin{array}{cc} \left\{0\right\}, & t\in \left[0,0.4\right),\\ \left\{0\right\}\cup A_{1}, & t\in \left[0.4,0.7\right),\\ A, & t\in \left[0.7,1\right], \end{array} \end{array}$$

and then *ξ*(*t*) is an ideal of *A* for any *t* ∈ [0,1]. Thus $\tilde{H}$ is a falling fuzzy ideal of *A*, where $\tilde{H}(x)=P(t|x\in \xi (t))$ is represented as follows:

(10) $$\begin{array}{c} \tilde{H}\left(x\right)=\{\begin{array}{cc} 0.3, & x\in A_{2}\cup \left\{1\right\},\\ 0.6, & x\in A_{1},\\ 1, & x=0. \end{array} \end{array}$$

Let (*Ω*, *A*, *P*) be a probability space and *F*(*A*): = {*f*∣*f* : *Ω* → *A*}. Define the operations ⊞ and ~ on *F*(*A*) as follows: ∀*ω* ∈ *Ω*, *f*, *g* ∈ *F*(*A*),

(11) $$\begin{array}{c} \left(f⊞g\right)\left(\omega \right)=f\left(\omega \right)⊕g\left(\omega \right),\;\;\left(\sim f\right)\left(\omega \right)=\neg f\left(\omega \right). \end{array}$$

Let 0 ∈ *F*(*A*) be defined by 0(*ω*) = 0 for any *ω* ∈ *Ω*. Then it is easy to check that (*F*(*A*), ⊞, ~, 0) is an MV-algebra. Define the operation ⊟ on *F*(*A*) by (*f*⊟*g*)(*ω*) = *f*(*ω*) ⊖ *g*(*ω*), for any *ω* ∈ *Ω*, *f*, *g* ∈ *F*(*A*).

For any subset *S* of *A* and *f* ∈ *F*(*A*), let *S*_*f*_ : = {*ω* ∈ *Ω*∣*f*(*ω*) ∈ *S*} and

(12) $$\begin{array}{c} \xi :\Omega ⟶P\left(F\left(A\right)\right),\\ \omega ⟼\left\{f\in F\left(\omega \right)\;|\;f\left(\omega \right)\in S\right\}. \end{array}$$

Then *S*_*f*_ ∈ *A*.

**Proposition 18 .**

Let (*Ω*, *A*, *P*) be a probability space, *S* a nonempty subset of *A*, and *ω* ∈ *Ω*. If *S* is an ideal of *A*, then *ξ*(*ω*) = {*f* ∈ *F*(*A*)∣*f*(*ω*) ∈ *S*} is an ideal of *F*(*A*).

**Proof**

Suppose that *S* is an ideal of *A*. Since 0(*ω*) = 0 ∈ *S* for any *ω* ∈ *Ω*, we have that 0 ∈ *ξ*(*ω*). Let *f*, *g* ∈ *ξ*(*ω*) be such that *f*⊟*g* ∈ *ξ*(*ω*) and *g* ∈ *ξ*(*ω*). For any *ω* ∈ *Ω*, we have *f*(*ω*) ⊖ *g*(*ω*) = (*f*⊟*g*)(*ω*) ∈ *A* and *g*(*ω*) ∈ *S*. Hence *f*(*ω*) ∈ *S*; that is, *f* ∈ *ξ*(*ω*). Thus *ξ*(*ω*) is an ideal of *F*(*A*).

Noticing that *ξ* ^−1^(*f*) = {*ω* ∈ *Ω*∣*f* ∈ *ξ*(*ω*)} = {*ω* ∈ *Ω*∣*f*(*ω*) ∈ *S*} = *S*_*f*_ ∈ *A* and *ξ* is a random set of *F*(*A*), we get that $\tilde{H}$ is a falling fuzzy ideal of *F*(*A*), where $\tilde{H}(f)=P(\omega |f(\omega )\in S)$.

**Proposition 19 .**

Let *μ* be a *T*_∧_-fuzzy ideal of *A*. Then *μ* is a falling fuzzy ideal of *A*.

**Proof**

Consider the probability space (*Ω*, *A*, *P*) = ([0,1], *A*, *m*), where *A* is a Borel field on [0,1] and *m* is the usual Lebesgue measure. Since *μ* is a *T*_∧_-fuzzy ideal of *A*, then *L*(*μ*; *t*) is an ideal of *A* for any *t* ∈ [0,1], by Proposition 9. Define the random set *ξ* : [0,1] → *P*(*A*) by *ξ*(*t*) = *L*(*μ*; *t*) for any *t* ∈ [0,1]; then *μ* is a falling fuzzy ideal of *A*.

However, it is important and interesting to point out that the converse of Proposition 19 is not true in general and we illustrate it by the following example.

**Example 20 .**

Let *E* = {*a*, *b*, *c*} and *P*(*E*) be the power set of *E*. Let ⊕, ¬, and 0 denote, respectively, the join, the complement, and the smallest element in *A* : = *P*(*E*). It is clear that (*A*, ⊕, ¬, 0) is an MV-algebra.

Let (*Ω*, *A*, *P*) = ([0,1], *A*, *m*), where *A* is a Borel field on [0,1] and *m* is the usual Lebesgue measure. The mapping *ξ* : *Ω* → *P*(*A*) is defined by

(13) $$\begin{array}{c} \xi \left(t\right)=\{\begin{array}{cc} \left\{\emptyset \right\}, & t\in \left[0,0.2\right),\\ \left\{\emptyset ,\left\{a\right\}\right\}, & t\in \left[0.2,0.7\right),\\ \left\{\emptyset ,\left\{c\right\},\left\{b\right\},\left\{b,c\right\}\right\}, & t\in \left[0.7,0.8\right),\\ A, & t\in \left[0.8,1\right]. \end{array} \end{array}$$

Then *ξ*(*t*) is an ideal of *A* for any *t* ∈ [0,1]. Thus $\tilde{H}$ is a falling fuzzy ideal of *A*, where $\tilde{H}(x)=P(t|x\in \xi (t))$ is represented as follows:

(14) $$\begin{array}{c} \tilde{H}\left(x\right)=\{\begin{array}{cc} 0.2, & x=\left\{a,c\right\},\left\{a,b\right\},\left\{a,b,c\right\},\\ 0.3, & x=\left\{c\right\},\left\{b\right\},\left\{b,c\right\},\\ 0.7, & x=\left\{a\right\},\\ 1, & x=\emptyset . \end{array} \end{array}$$

But $\tilde{H}$ is not a *T*_∧_-fuzzy ideal of *A* since $\tilde{H}(\{a\}⊕\{c\})=\tilde{H}(\{a,c\})=0.2<\min\{\tilde{H}(\{a\}),\tilde{H}(\{c\})\}=0.3$.

Let (*Ω*, *A*, *P*) be a probability space and $\tilde{H}$ a falling shadow of a random set *ξ* : *Ω* → *P*(*A*). For any *x* ∈ *A*, let *Ω*(*x*; *ξ*) = {*ω* ∈ *Ω*∣*x* ∈ *ξ*(*ω*)}; then *Ω*(*x*; *ξ*) ∈ *A*. In what follows, we give a number of equivalent conditions of falling fuzzy ideals for further discussion.

**Theorem 21 .**

Let $\tilde{H}$ be a falling shadow of a random set *ξ* : *Ω* → *P*(*A*). Then $\tilde{H}$ is a falling fuzzy ideal of *A* if and only if (1)  0 ∈ *ξ*(*ω*), (2)  *y* ∈ *ξ*(*ω*) and *x* ∈ *A*∖*ξ*(*ω*) imply *x* ⊖ *y* ∈ *A*∖*ξ*(*ω*) for any *ω* ∈ *Ω*, *x*, *y* ∈ *A*.

**Proof**

Assuming that $\tilde{H}$ is a falling fuzzy ideal of *A*, then *ξ*(*ω*) is an ideal of *A* for any *ω* ∈ *Ω*, and it follows that 0 ∈ *ξ*(*ω*). Let *x*, *y* ∈ *A* be such that *y* ∈ *ξ*(*ω*) and *x* ∈ *A*∖*ξ*(*ω*). Supposing that *x* ⊖ *y* ∈ *ξ*(*ω*), then *x* ∈ *ξ*(*ω*), a contradiction, and thus (2) is valid.

Conversely, assume that (1) and (2) are valid. For any *ω* ∈ *Ω*, let *x*, *y* ∈ *A* be such that *x* ⊖ *y* ∈ *ξ*(*ω*) and *y* ∈ *ξ*(*ω*). Supposing that *x* ∈ *A*∖*ξ*(*ω*), it follows that *x* ⊖ *y* ∈ *A*∖*ξ*(*ω*), which is a contradiction, and thus *x* ∈ *ξ*(*ω*). Hence $\tilde{H}$ is a falling fuzzy ideal of *A*.

**Theorem 22 .**

Let *ξ* : *Ω* → *P*(*A*) be a random set and $\tilde{H}$ a falling shadow of *ξ*. Then $\tilde{H}$ is a falling fuzzy ideal of *A* if and only if *z* ⊖ *x* ≤ *y* implies *Ω*(*x*; *ξ*)∩*Ω*(*y*; *ξ*)⊆*Ω*(*z*; *ξ*) for any *x*, *y*, *z* ∈ *A*.

**Proof**

Let *x*, *y*, *z* ∈ *A* be such that *z* ⊖ *x* ≤ *y*. For any *ω* ∈ *Ω*(*x*; *ξ*)∩*Ω*(*y*; *ξ*), we get *x* ∈ *ξ*(*ω*), *y* ∈ *ξ*(*ω*). Considering that $\tilde{H}$ is a falling fuzzy ideal of *A*, we have that *ξ*(*ω*) is an ideal of *A*. By Proposition 6, *z* ∈ *ξ*(*ω*); that is, *ω* ∈ *Ω*(*z*; *ξ*). Hence *Ω*(*x*; *ξ*)∩*Ω*(*y*; *ξ*)⊆*Ω*(*z*; *ξ*).

Conversely, assume that *z* ⊖ *x* ≤ *y* implies *Ω*(*x*; *ξ*)∩*Ω*(*y*; *ξ*)⊆*Ω*(*z*; *ξ*) for any *x*, *y*, *z* ∈ *A*. Let *ω* ∈ *Ω* be such that *ξ*(*ω*) ≠ *∅*. Then there exists *x* ∈ *ξ*(*ω*); that is, *ω* ∈ *Ω*(*x*; *ξ*). Since 0 ⊖ *x* = 0 ≤ *x*, then *Ω*(*x*; *ξ*)∩*Ω*(*x*; *ξ*) = *Ω*(*x*; *ξ*)⊆*Ω*(0; *ξ*). It follows that *ω* ∈ *Ω*(0; *ξ*); that is, 0 ∈ *ξ*(*ω*). Let *x*, *y* ∈ *A* be such that *x* ⊖ *y* ∈ *ξ*(*ω*) and *y* ∈ *ξ*(*ω*). Then *ω* ∈ *Ω*(*x* ⊖ *y*; *ξ*)∩*Ω*(*y*; *ξ*). Since *x* ⊖ (*x* ⊖ *y*) ≤ *y*, it follows that *Ω*(*x* ⊖ *y*; *ξ*)∩*Ω*(*y*; *ξ*)⊆*Ω*(*x*; *ξ*). Thus *ω* ∈ *Ω*(*x*; *ξ*); that is, *x* ∈ *ξ*(*ω*). Hence *ξ*(*ω*) is an ideal of *A*, and so $\tilde{H}$ is a falling fuzzy ideal of *A*.

**Lemma 23 .**

Let *ξ* : *Ω* → *P*(*A*) be a random set and $\tilde{H}$ a falling shadow of *ξ*. If $\tilde{H}$ is a falling fuzzy ideal of *A*, then the following statements hold: for any *x*, *y* ∈ *A*,

1. *Ω*(*x*; *ξ*)⊆*Ω*(0; *ξ*);
2. *Ω*(*x* ⊖ *y*; *ξ*)∩*Ω*(*y*; *ξ*)⊆*Ω*(*x*; *ξ*).

**Proof**

1. Noticing that $\tilde{H}$ is a falling fuzzy ideal of *A*, we have that *ξ*(*ω*) is an ideal of *A* for any *ω* ∈ *Ω*(*x*; *ξ*). It follows that 0 ∈ *ξ*(*ω*); that is, *ω* ∈ *Ω*(0; *ξ*). Hence *Ω*(*x*; *ξ*)⊆*Ω*(0; *ξ*).
2. For any *ω* ∈ *Ω*(*x* ⊖ *y*; *ξ*)∩*Ω*(*y*; *ξ*), we get *x* ⊖ *y* ∈ *ξ*(*ω*) and *y* ∈ *ξ*(*ω*). Since *ξ*(*ω*) is an ideal of *A*, then *x* ∈ *ξ*(*ω*); that is, *ω* ∈ *Ω*(*x*; *ξ*). Thus *Ω*(*x* ⊖ *y*; *ξ*)∩*Ω*(*y*; *ξ*)⊆*Ω*(*x*; *ξ*).

**Theorem 24 .**

Let $\tilde{H}$ be a falling shadow of a random set *ξ* : *Ω* → *P*(*A*). Then $\tilde{H}$ is a falling fuzzy ideal of *A* if and only if, for any *x*, *y* ∈ *A*,

1. *Ω*(*x*; *ξ*)∩*Ω*(*y*; *ξ*)⊆*Ω*(*x* ⊕ *y*; *ξ*);
2. *x* ≤ *y* implies *Ω*(*y*; *ξ*)⊆*Ω*(*x*; *ξ*).

**Proof**

Assuming that $\tilde{H}$ is a falling fuzzy ideal of *A*, then *ξ*(*ω*) is an ideal of *A* for any *ω* ∈ *Ω*. For any *x*, *y* ∈ *A*, if *x* ∈ *ξ*(*ω*), *y* ∈ *ξ*(*ω*), then *x* ⊕ *y* ∈ *ξ*(*ω*). That is, *ω* ∈ *Ω*(*x*; *ξ*) and *ω* ∈ *Ω*(*y*; *ξ*) imply *ω* ∈ *Ω*(*x* ⊕ *y*; *ξ*). Hence *Ω*(*x*; *ξ*)∩*Ω*(*y*; *ξ*)⊆*Ω*(*x* ⊕ *y*; *ξ*). If *x* ≤ *y*, that is, *x* ⊖ 0 ≤ *y*, it follows that *Ω*(*y*; *ξ*)⊆*Ω*(*x*; *ξ*) by Theorem 22 and Lemma 23.

Conversely, assume that (1) and (2) are valid. For any *ω* ∈ *Ω*, let *x*, *y* ∈ *A* be such that *x* ∈ *ξ*(*ω*) and *y* ∈ *ξ*(*ω*). It follows that *ω* ∈ *Ω*(*x*; *ξ*)∩*Ω*(*y*; *ξ*). Thus *ω* ∈ *Ω*(*x* ⊕ *y*; *ξ*); that is, *x* ⊕ *y* ∈ *ξ*(*ω*). If *x* ≤ *y* and *y* ∈ *ξ*(*ω*) for any *x*, *y* ∈ *A*, then *ω* ∈ *Ω*(*y*; *ξ*)⊆*Ω*(*x*; *ξ*). Thus *x* ∈ *ξ*(*ω*). Hence *ξ*(*ω*) is an ideal of *A*, and so $\tilde{H}$ is a falling fuzzy ideal of *A*.

**Proposition 25 .**

Let $\tilde{H}$ be a falling shadow of a random set *ξ* : *Ω* → *P*(*A*). Then $\tilde{H}$ is a falling fuzzy ideal of *A* if and only if, for any *x*, *y* ∈ *A*,

1. *Ω*(*x*; *ξ*)∩*Ω*(*y*; *ξ*)⊆*Ω*(*x* ⊕ *y*; *ξ*);
2. *Ω*(*y*; *ξ*)⊆*Ω*(*x*∧*y*; *ξ*).

**Proof**

The proof is obvious since *x* ≤ *y* if and only if *x*∧*y* = *x* for any *x*, *y* ∈ *A*.

**Proposition 26 .**

Let $\tilde{H}$ be a falling shadow of a random set *ξ* : *Ω* → *P*(*A*). Then $\tilde{H}$ is a falling fuzzy ideal of *A* if and only if, for any *x*, *y* ∈ *A*,

1. *Ω*(*x*; *ξ*)∩*Ω*(*y*; *ξ*)⊆*Ω*(*x* ⊕ *y*; *ξ*);
2. *Ω*(*y*; *ξ*)⊆*Ω*(*x* ⊗ *y*; *ξ*).

**Proof**

One direction is clear since *x* ⊗ *y* ≤ *y*. Conversely, assume that conditions (1) and (2) hold. By hypothesis, we have *Ω*(*y*; *ξ*)⊆*Ω*(*y* ⊗ (¬*y* ⊕ *x*); *ξ*) for any *x*, *y* ∈ *A*. Now let *x* ≤ *y* and *ω* ∈ *Ω*(*y*; *ξ*). Then *y* ∈ *ξ*(*ω*) and *y* ⊗ (¬*y* ⊕ *x*) = *x*∧*y* = *x* ∈ *ξ*(*ω*). It means that *x* ≤ *y* implies *Ω*(*y*; *ξ*)⊆*Ω*(*x*; *ξ*), and so $\tilde{H}$ is a falling fuzzy ideal of *A* by Theorem 24.

**Theorem 27 .**

Let $\tilde{H}$ be a falling shadow of a random set *ξ* : *Ω* → *P*(*A*). If the following conditions are valid:

1. *Ω* = *Ω*(0; *ξ*);
2. *Ω*(*x* ⊖ *y*; *ξ*)∩*Ω*(*y*; *ξ*)⊆*Ω*(*x*; *ξ*), for any *x*, *y* ∈ *A*,

then $\tilde{H}$ is a falling fuzzy ideal of *A*.

**Proof**

Assume that conditions (1) and (2) are valid. For any *ω* ∈ *Ω*, we have *ω* ∈ *Ω*(0; *ξ*); that is, 0 ∈ *ξ*(*ω*). For any *x*, *y* ∈ *A*, if *x* ⊖ *y* ∈ *ξ*(*ω*) and *y* ∈ *ξ*(*ω*), then *ω* ∈ *Ω*(*x* ⊖ *y*; *ξ*), *ω* ∈ *Ω*(*y*; *ξ*). It follows that *ω* ∈ *Ω*(*x* ⊖ *y*; *ξ*)∩*Ω*(*y*; *ξ*)⊆*Ω*(*x*; *ξ*). Hence *ω* ∈ *Ω*(*x*; *ξ*); that is, *x* ∈ *ξ*(*ω*). Thus *ξ*(*ω*) is an ideal of *A*, and so $\tilde{H}$ is a falling fuzzy ideal of *A*.

**Proposition 28 .**

Let $\tilde{H}$ be a falling shadow of a random set *ξ* : *Ω* → *P*(*A*). If $\tilde{H}$ is a falling fuzzy ideal of *A*, then the following relationships hold: for any *x*, *y*, *z* ∈ *A*,

1. *Ω*(*x* ⊖ *z*; *ξ*)∩*Ω*(*z* ⊖ *y*; *ξ*)⊆*Ω*(*x* ⊖ *y*; *ξ*);
2. if *Ω*(*x* ⊖ *y*; *ξ*) = *Ω*(0; *ξ*), then *Ω*(*y*; *ξ*)⊆*Ω*(*x*; *ξ*);
3. *Ω*((*x* ⊖ *y*) ⊖ *z*; *ξ*)∩*Ω*(*y* ⊖ *z*; *ξ*)⊆*Ω*((*x* ⊖ *z*) ⊖ *z*; *ξ*);
4. *Ω*(*x* ⊖ *y*; *ξ*)∩*Ω*(*y*; *ξ*) = *Ω*(*x*; *ξ*)∩*Ω*(*y*; *ξ*);
5. *Ω*(1 ⊖ *y*; *ξ*)∩*Ω*(*y*; *ξ*) = *Ω*(1; *ξ*);
6. if *x* ≤ *y*, then *Ω*(*y* ⊖ *x*; *ξ*)∩*Ω*(*x*; *ξ*) = *Ω*(*y*; *ξ*).

**Proof**

Here we only prove (3), and the other cases directly follow from Lemma 23 and Theorem 24. For any *x*, *y*, *z* ∈ *A*, we have *Ω*((*x* ⊖ *z*) ⊖ *z*; *ξ*)⊇*Ω*((*x* ⊖ *z*) ⊖ *y*; *ξ*)∩*Ω*(*y* ⊖ *z*; *ξ*) = *Ω*((*x* ⊖ *y*) ⊖ *z*; *ξ*)∩*Ω*(*y* ⊖ *z*; *ξ*) by (1).

According to Proposition 28, we can provide another condition for a falling shadow to be a falling fuzzy ideal in MV-algebras.

**Proposition 29 .**

Let $\tilde{H}$ be a falling shadow of a random set *ξ* : *Ω* → *P*(*A*). If the following conditions are valid:

1. *Ω* = *Ω*(0; *ξ*);
2. *Ω*((*x* ⊖ *y*) ⊖ *z*; *ξ*)∩*Ω*(*y* ⊖ *z*; *ξ*)⊆*Ω*((*x* ⊖ *z*) ⊖ *z*; *ξ*), for any *x*, *y*, *z* ∈ *A*,

then $\tilde{H}$ is a falling fuzzy ideal of *A*.

**Proof**

By hypothesis, we get *Ω*(*x* ⊖ *y*; *ξ*)∩*Ω*(*y*; *ξ*) = *Ω*((*x* ⊖ *y*) ⊖ 0; *ξ*)∩*Ω*(*y* ⊖ 0; *ξ*)⊆*Ω*((*x* ⊖ 0) ⊖ 0; *ξ*) = *Ω*(*x*; *ξ*), for any *x*, *y* ∈ *A*. It follows from Theorem 27 that $\tilde{H}$ is a falling fuzzy ideal of *A*.

From Proposition 19, it is known that the notion of falling fuzzy ideals is a generalization of that of *T*_∧_-fuzzy ideals. Under what conditions a falling fuzzy ideal becomes a *T*-fuzzy ideal, we will give some answers to the questions in the following.

**Theorem 30 .**

Let $\tilde{H}$ be a falling shadow of a random set *ξ* : *Ω* → *P*(*A*). If $\tilde{H}$ is a falling fuzzy ideal of *A*, then $\tilde{H}$ is a *T*_*m*_-fuzzy ideal of *A*.

**Proof**

Noticing that $\tilde{H}$ is a falling fuzzy ideal of *A*, we have that *Ω*(*x*; *ξ*)⊆*Ω*(0; *ξ*) for any *x* ∈ *A*, by Lemma 23. Since $\tilde{H}(0)=P(\omega |0\in \xi (\omega ))\geq P(\omega |x\in \xi (\omega ))=\tilde{H}(x)$, thus $\tilde{H}(0)\geq \tilde{H}(x)$. It follows that *Ω*(*x* ⊖ *y*; *ξ*)∩*Ω*(*y*; *ξ*)⊆*Ω*(*x*; *ξ*) for any *x*, *y* ∈ *A* by Lemma 23; that is, {*ω* ∈ *Ω*∣*x* ⊖ *y* ∈ *ξ*(*ω*)}∩{*ω* ∈ *Ω*∣*y* ∈ *ξ*(*ω*)}⊆{*ω* ∈ *Ω*∣*x* ∈ *ξ*(*ω*)}. Thus $\tilde{H}(x)=P(\omega |x\in \xi (\omega ))\geq P(\{\omega |x⊖y\in \xi (\omega )\}\cap \{\omega |y\in \xi (\omega )\})\geq P(\omega |x⊖y\in \xi (\omega ))+P(\omega |y\in \xi (\omega ))$  −  *P*(*ω*∣*x* ⊖ *y* ∈ *ξ*(*ω*) or $y\in \xi (\omega ))\geq \tilde{H}(x⊖y)+\tilde{H}(y)-1$. Since $\tilde{H}(x)\geq 0$, then $\tilde{H}(x)\geq T_{m}(\tilde{H}(x⊖y),\tilde{H}(y))$. Hence $\tilde{H}$ is a *T*_*m*_-fuzzy ideal of *A*.

**Proposition 31 .**

Let $\tilde{H}$ be a falling shadow of a random set *ξ* : *Ω* → *P*(*A*). If $\tilde{H}$ is a falling fuzzy ideal of *A*, then the following statements hold: ∀*x*, *y* ∈ *A*,

1. if *Ω*(*x*; *ξ*)⊆*Ω*(*y*; *ξ*) or *Ω*(*y*; *ξ*)⊆*Ω*(*x*; *ξ*), then $\tilde{H}$ is a *T*_∧_-fuzzy ideal of *A*;
2. if *Ω*(*x*; *ξ*) and *Ω*(*y*; *ξ*) are independent random events, then $\tilde{H}$ is a *T*_*p*_-fuzzy ideal of *A*.

**Proof**

1. By Theorem 30, $\tilde{H}(0)\geq \tilde{H}(x)$ for any *x* ∈ *A*. According to hypothesis and Lemma 23, we have *Ω*(*x* ⊖ *y*; *ξ*)⊆*Ω*(*x*; *ξ*) or *Ω*(*y*; *ξ*)⊆*Ω*(*x*; *ξ*) for any *x*, *y* ∈ *A*. If *Ω*(*x* ⊖ *y*; *ξ*)⊆*Ω*(*x*; *ξ*), then $\tilde{H}(x)=P(\omega |x\in \xi (\omega ))\geq P(\{\omega |x⊖y\in \xi (\omega )\}=\tilde{H}(x⊖y)$. If *Ω*(*y*; *ξ*)⊆*Ω*(*x*; *ξ*), then $\tilde{H}(x)=P(\omega |x\in \xi (\omega ))\geq P(\{\omega |y\in \xi (\omega )\}=\tilde{H}(y)$. Hence $\tilde{H}(x)\geq \min\{\tilde{H}(x⊖y),\tilde{H}(y)\}$, and so $\tilde{H}$ is a *T*_∧_-fuzzy ideal of *A*.
2. It is directly obtained that $\tilde{H}(0)\geq \tilde{H}(x)$ for any *x* ∈ *A*. Since $\tilde{H}$ is a falling fuzzy ideal of *A*, then {*ω* ∈ *Ω*∣*x* ⊖ *y* ∈ *ξ*(*ω*)}∩{*ω* ∈ *Ω*∣*y* ∈ *ξ*(*ω*)}⊆{*ω* ∈ *Ω*∣*x* ∈ *ξ*(*ω*)}, for any *x*, *y* ∈ *A*. It follows that $\tilde{H}(x)=P(\omega |x\in \xi (\omega ))\geq P(\{\omega |x⊖y\in \xi (\omega )\}\cap \{\omega |y\in \xi (\omega )\})=\tilde{H}(x⊖y)\tilde{H}(y)$. Thus $\tilde{H}$ is a *T*_*p*_-fuzzy ideal of *A*.

**Definition 32 .**

Let (*Ω*, *A*, *P*) be a probability space, and let $\tilde{H}$, ${\tilde{H}}_{1}$, and ${\tilde{H}}_{2}$ be falling shadows of random sets *ξ*, *ξ*_1_, *ξ*_2_ : *Ω* → *P*(*A*), respectively. Then the product of ${\tilde{H}}_{1}$ and ${\tilde{H}}_{2}$ is defined by

(15) (H~1⊛H~2)(z) =sup⁡{P([0,H~1(x)]×[0,H~2(y)]) ∣ z=x⊕y}.

The up product between ${\tilde{H}}_{1}$ and ${\tilde{H}}_{2}$ is defined by

(16) (H~1⊚H~2)(z) =sup⁡{P([0,H~1(x)]×[0,H~2(y)]) ∣ z≤x⊕y}.

The upset of $\tilde{H}$ is defined by

(17) $$\begin{array}{c} {\tilde{H}}^{↑}\left(z\right)=\sup\left\{P\left(\left[0,\tilde{H}\left(x\right)\right]\right)\;|\;z\leq x\right\}. \end{array}$$

From the above definition, it is easy to see that ${\tilde{H}}^{↑}(z)=\sup\{P([0,\tilde{H}(x)])|z\leq x\}=\sup\{\tilde{H}(x)|z\leq x\}\geq \tilde{H}(z)$ for any *z* ∈ *A*, and so $\tilde{H}\subseteq {\tilde{H}}^{↑}$.

If the probability distribution *P* in Definition 32 is diagonal, antidiagonal, and independent, respectively, then $\left({\tilde{H}}_{1}⊛{\tilde{H}}_{2})(z)=\sup\{T({\tilde{H}}_{1}(x),{\tilde{H}}_{2}(y))|z=x⊕y\right\}$ and $\left({\tilde{H}}_{1}⊚{\tilde{H}}_{2})(z)=\sup\{T({\tilde{H}}_{1}(x),{\tilde{H}}_{2}(y))|z\leq x⊕y\right\}$, where *T* ∈ {*T*_∧_, *T*_*m*_, *T*_*p*_}.

**Proposition 33 .**

Let (*Ω*, *A*, *P*) be a probability space and $\tilde{H}$ a falling shadow of a random set *ξ* : *Ω* → *P*(*A*). Then $\tilde{H}={\tilde{H}}^{↑}$ if and only if *x* ≤ *y* implies $\tilde{H}(y)\leq \tilde{H}(x)$ for any *x*, *y* ∈ *A*.

**Proof**

Suppose that $\tilde{H}={\tilde{H}}^{↑}$. For any *x*, *y* ∈ *A*, if *x* ≤ *y*, then $\tilde{H}(x)={\tilde{H}}^{↑}(x)=\sup\{\tilde{H}(z)|x\leq z\}\geq \tilde{H}(y)$.

Conversely, for any *z* ∈ *A*, we have ${\tilde{H}}^{↑}(z)=\sup\{\tilde{H}(x)|z\leq x\}\leq \tilde{H}(z)$ by hypothesis. Thus ${\tilde{H}}^{↑}\subseteq \tilde{H}$, and so $\tilde{H}={\tilde{H}}^{↑}$.

**Theorem 34 .**

Let (*Ω*, *A*, *P*) be a probability space and $\tilde{H}$ a falling shadow of a random set *ξ* : *Ω* → *P*(*A*). If the probability distribution *P* of two-dimensional random variables is diagonal (antidiagonal or independent, resp.), then $\tilde{H}$ is a *T*-fuzzy ideal of *A* if and only if

1. $(\tilde{H}⊛\tilde{H})\subseteq \tilde{H}$,
2. ${\tilde{H}}^{↑}\subseteq \tilde{H}$,

where *T* ∈ {*T*_∧_, *T*_*m*_, *T*_*p*_}.

**Proof**

We only consider that *P* is diagonal, and other cases can be proved similarly. Supposing that $\tilde{H}$ is a *T*_∧_-fuzzy ideal of *A*, there exist *x*, *y* ∈ *A* such that *z* = *x* ⊕ *y* for any *z* ∈ *A*, then we get that $\tilde{H}(z)=\tilde{H}(x⊕y)\geq T_{\wedge }(\tilde{H}(x),\tilde{H}(y))$, and so $\tilde{H}(z)\geq \sup\{T_{\wedge }(\tilde{H}(x),\tilde{H}(y))|z=x⊕y\}=(\tilde{H}⊛\tilde{H})(z)$. Thus $(\tilde{H}⊛\tilde{H})\subseteq \tilde{H}$. For any *z* ∈ *A*, there exists *x* ∈ *A* such that *z* ≤ *x*, then $\tilde{H}(z)\geq \tilde{H}(x)$, and thus $\tilde{H}(z)\geq \sup\{\tilde{H}(x)|z\leq x\}={\tilde{H}}^{↑}(x)$. Therefore, ${\tilde{H}}^{↑}\subseteq \tilde{H}$.

Conversely, assume that $(\tilde{H}⊛\tilde{H})\subseteq \tilde{H}$ and ${\tilde{H}}^{↑}\subseteq \tilde{H}$ hold. For any *x*, *y* ∈ *A*, we have $\tilde{H}(x⊕y)\geq (\tilde{H}⊛\tilde{H})(x⊕y)\geq T_{\wedge }(\tilde{H}(x),\tilde{H}(y))$. If *x* ≤ *y*, then $\tilde{H}(x)\geq {\tilde{H}}^{↑}(x)\geq \tilde{H}(y)$. Therefore, $\tilde{H}$ is a *T*_∧_-fuzzy ideal of *A*.

**Theorem 35 .**

Let (*Ω*, *A*, *P*) be a probability space and $\tilde{H}$ a falling shadow of a random set *ξ* : *Ω* → *P*(*A*). If the probability distribution *P* of two-dimensional random variables is diagonal, then $\tilde{H}$ is a *T*_∧_-fuzzy ideal of *A* if and only if $(\tilde{H}⊚\tilde{H})\subseteq \tilde{H}$.

**Proof**

Suppose that $\tilde{H}$ is a *T*_∧_-fuzzy ideal of *A*. For any *z* ∈ *A*, there exist *x*, *y* ∈ *A* such that *z* ≤ *x* ⊕ *y*, then we have $\tilde{H}(z)\geq T_{\wedge }(\tilde{H}(x),\tilde{H}(y))$, and so $\tilde{H}(z)\geq \sup\{T_{\wedge }(\tilde{H}(x),\tilde{H}(y))|z\leq x⊕y\}=(\tilde{H}⊚\tilde{H})(z)$. Thus $(\tilde{H}⊚\tilde{H})\subseteq \tilde{H}$.

Conversely, assume that $(\tilde{H}⊚\tilde{H})\subseteq \tilde{H}$. For any *x* ∈ *A*, we have 0 ≤ *x* ⊕ 0, and so $\tilde{H}(0)\geq (\tilde{H}⊚\tilde{H})(0)\geq T_{\wedge }(\tilde{H}(x),\tilde{H}(x))=\tilde{H}(x)$. For any *x*, *y* ∈ *A*, *x* ≤ *y* ⊕ (*x* ⊖ *y*), and then $\tilde{H}(x)\geq (\tilde{H}⊚\tilde{H})(x)\geq T_{\wedge }(\tilde{H}(y),\tilde{H}(x⊖y))$. Therefore, $\tilde{H}$ is a *T*_∧_-fuzzy ideal of *A*.

**Proposition 36 .**

Let (*Ω*, *A*, *P*) be a probability space and $\tilde{H}$ a falling shadow of a random set *ξ* : *Ω* → *P*(*A*). If $\tilde{H}(0)=1$ and the probability distribution *P* of two-dimensional random variables is antidiagonal (or independent), then $\tilde{H}$ is a *T*_*m*_-fuzzy ideal (or *T*_*p*_-fuzzy ideal) of *A* if and only if $(\tilde{H}⊚\tilde{H})\subseteq \tilde{H}$.

**Proof**

We only consider that *P* is antidiagonal. Suppose that $(\tilde{H}⊚\tilde{H})\subseteq \tilde{H}$. For any *x* ∈ *A*, we have 0 ≤ *x* ⊕ 0, and so $\tilde{H}(0)\geq (\tilde{H}⊖\tilde{H})(0)\geq T_{m}(\tilde{H}(0),\tilde{H}(x))=T_{m}(1,\tilde{H}(x))=\tilde{H}(x)$. For any *x*, *y* ∈ *A*, *x* ≤ *y* ⊕ (*x* ⊖ *y*); then $\tilde{H}(x)\geq (\tilde{H}⊚\tilde{H})(x)\geq T_{m}(\tilde{H}(y),\tilde{H}(x⊖y))$. Therefore $\tilde{H}$ is a *T*_*m*_-fuzzy ideal of *A*.

Conversely, suppose that $\tilde{H}$ is a *T*_*m*_-fuzzy ideal of *A*. The proof of $(\tilde{H}⊚\tilde{H})\subseteq \tilde{H}$ is similar to that of Theorem 35.

**Proposition 37 .**

Let (*Ω*, *A*, *P*) be a probability space, where the probability distribution *P* of two-dimensional random variables is diagonal. Let ${\tilde{H}}_{1}$ and ${\tilde{H}}_{2}$ be falling shadows of random sets *ξ*_1_, *ξ*_2_ : *Ω* → *P*(*A*), respectively. If ${\tilde{H}}_{1}$ and ${\tilde{H}}_{2}$ are *T*_∧_-fuzzy ideals of *A*, then ${\tilde{H}}_{1}⊚{\tilde{H}}_{2}$ is a *T*_∧_-fuzzy ideal of *A*.

**Proof**

Since ${\tilde{H}}_{1}$ and ${\tilde{H}}_{2}$ are *T*_∧_-fuzzy ideals of *A*, then we have

(18) (H~1⊚H~2)(x⊕y) ≥sup⁡{H~1(x1⊕y1)∧H~2(x2⊕y2) ∣ x≤x1⊕x2,y≤y1⊕y1} ≥sup⁡⁡{H~1(x1)∧H~1(y1)∧H~2(x2)∧H~2(y2) ∣ x≤x1⊕x2,y≤y1⊕y1} =sup⁡{H~1(x1)∧H~2(x2) ∣ x≤x1⊕x2}  ∧sup⁡{H~1(y1)∧H~2(y2) ∣ y≤y1⊕y1} =(H~1⊚H~2)(x)∧(H~1⊚H~2)(y)

for any *x*, *y* ∈ *A*.

If *x* ≤ *y*, then $\left({\tilde{H}}_{1}⊚{\tilde{H}}_{2})(x)\geq \sup\{{\tilde{H}}_{1}(x_{1})\wedge {\tilde{H}}_{2}(x_{2})|x\leq x_{1}⊕x_{2}\}\geq \sup\{{\tilde{H}}_{1}(y_{1})\wedge {\tilde{H}}_{2}(y_{2})|x\leq y\leq y_{1}⊕y_{1}\right\}$ = $\left({\tilde{H}}_{1}⊚{\tilde{H}}_{2})(y\right)$. Therefore ${\tilde{H}}_{1}⊚{\tilde{H}}_{2}$ is a *T*_∧_-fuzzy ideal of *A*.

## 4. Characterizations of Falling Fuzzy Implicative Ideals

In the section, we introduce the notion of falling fuzzy implicative ideals as a generalization of *T*_∧_-fuzzy implicative ideals in MV-algebras and investigate some of their properties. We also give some conditions under which falling shadows (falling fuzzy ideals) become falling fuzzy implicative ideals.

**Definition 38 .**

A fuzzy set *μ* in *A* is called a *T*-fuzzy implicative ideal of *A* if it satisfies ∀*x*, *y*, *z* ∈ *A*,

1. *μ*(0) ≥ *μ*(*x*);
2. *μ*(*x* ⊖ *z*) ≥ *T*(*μ*((*x* ⊖ *y*) ⊖ *z*), *μ*(*y* ⊖ *z*)).

**Definition 39 .**

Let (*Ω*, *A*, *P*) be a probability space and *ξ* : *Ω* → *P*(*A*) be a random set. If *ξ*(*ω*) is an implicative ideal of *A* for any *ω* ∈ *Ω*, then the falling shadow $\tilde{H}$ of *ξ*, that is, ∀*u* ∈ *A*,

(19) $$\begin{array}{c} \tilde{H}\left(u\right)=P\left(\omega \;|\;u\in \xi \left(\omega \right)\right), \end{array}$$

is called a falling fuzzy implicative ideal of *A*.

For the sake of simplicity and better understanding the above definition, we give the following example.

**Example 40 .**

Let *A* = {0, *a*, *b*, *c*, *d*, 1} be such that 0 < *b* < *a* < 1, 0 < *d* < *a* < 1, and 0 < *d* < *c* < 1. Define the operations ⊕ and ¬ on *A* as follows:

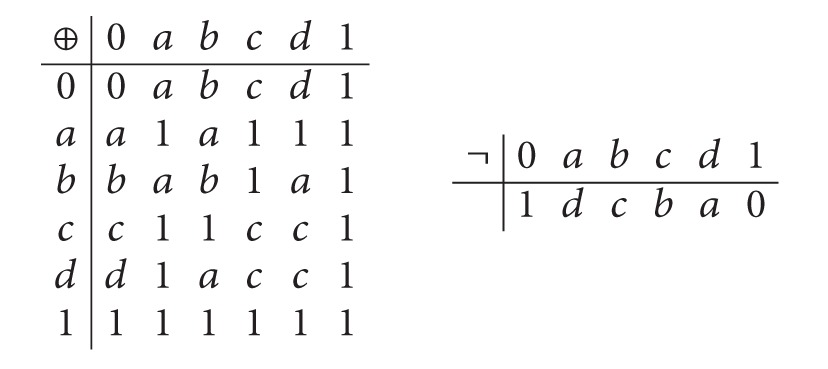
(20)

Routine computations prove that (*A*, ⊕, ¬, 0) is an MV-algebra.

Let (*Ω*, *A*, *P*) = ([0,1], *A*, *m*), where *A* is a Borel field on [0,1] and *m* is the usual Lebesgue measure. The mapping *ξ* : *Ω* → *P*(*A*) is defined by

(21) $$\begin{array}{c} \xi \left(t\right)=\{\begin{array}{cc} \left\{0\right\}, & t\in \left[0,0.3\right),\\ \left\{0,d\right\}, & t\in \left[0.3,0.5\right),\\ \left\{0,c,d\right\}, & t\in \left[0.5,0.7\right),\\ A, & t\in \left[0.7,1\right]. \end{array} \end{array}$$

Then *ξ*(*t*) is an implicative ideal of *A* for any *t* ∈ [0,1]. Thus $\tilde{H}$ is a falling fuzzy implicative ideal of *A*, where $\tilde{H}(x)=P(t|x\in \xi (t))$ is represented as follows:

(22) $$\begin{array}{c} \tilde{H}\left(x\right)=\{\begin{array}{cc} 0.3, & x=a,b,1,\\ 0.5, & x=c,\\ 0.7, & x=d,\\ 1, & x=0. \end{array} \end{array}$$

**Proposition 41 .**

Let (*Ω*, *A*, *P*) be a probability space, *S* a nonempty subset of *A*, and *ω* ∈ *Ω*. If *S* is an implicative ideal of *A*, then *ξ*(*ω*) = {*f* ∈ *F*(*A*)∣*f*(*ω*) ∈ *S*} is an implicative ideal of *F*(*A*).

**Proof**

It is similar to the proof of Proposition 18.

Since an implicative ideal is an ideal in MV-algebras and the converse is not true, thus we immediately get the following result.

**Proposition 42 .**

Every falling fuzzy implicative ideal of *A* is a falling fuzzy ideal, and the converse is not valid.

The following proposition describes the relationship between falling fuzzy implicative ideals and fuzzy implicative ideals, which is similar to Proposition 19, and we omit the proof.

**Proposition 43 .**

Let *μ* be a *T*_∧_-fuzzy implicative ideal of *A*. Then *μ* is a falling fuzzy implicative ideal of *A*.

According to Proposition 42 and Example 20, a falling fuzzy implicative ideal of *A* is a falling fuzzy ideal and a falling fuzzy ideal is not a *T*_∧_-fuzzy ideal of *A* in general, thus the converse of Proposition 43 is not true.

In what follows, we will display some characterizations of falling fuzzy implicative ideals.

**Theorem 44 .**

Let $\tilde{H}$ be a falling shadow of a random set *ξ* : *Ω* → *P*(*A*). Then $\tilde{H}$ is a falling fuzzy implicative ideal of *A* if and only if (1)  0 ∈ *ξ*(*ω*), (2)  *y* ⊖ *z* ∈ *ξ*(*ω*) and *x* ⊖ *z* ∈ *A*∖*ξ*(*ω*) imply (*x* ⊖ *y*) ⊖ *z* ∈ *A*∖*ξ*(*ω*) for any *ω* ∈ *Ω*, *x*, *y*, *z* ∈ *A*.

**Proof**

Suppose that $\tilde{H}$ is a falling fuzzy implicative ideal of *A*, it follows that *ξ*(*ω*) is an implicative ideal of *A* for any *ω* ∈ *Ω*, and thus 0 ∈ *ξ*(*ω*). Let *x*, *y*, *z* ∈ *A* be such that *y* ⊖ *z* ∈ *ξ*(*ω*) and *x* ⊖ *z* ∈ *A*∖*ξ*(*ω*). If (*x* ⊖ *y*) ⊖ *z* ∈ *ξ*(*ω*) hold, then *x* ⊖ *z* ∈ *ξ*(*ω*), which is a contradiction, and so (2) is valid.

Conversely, let *x*, *y*, *z* ∈ *A* be such that (*x* ⊖ *y*) ⊖ *z* ∈ *ξ*(*ω*) and *y* ⊖ *z* ∈ *ξ*(*ω*) for any *ω* ∈ *Ω*. If *x* ⊖ *z* ∈ *A*∖*ξ*(*ω*), it follows from hypothesis that (*x* ⊖ *y*) ⊖ *z* ∈ *A*∖*ξ*(*ω*), a contradiction, and so *x* ⊖ *z* ∈ *ξ*(*ω*). Therefore $\tilde{H}$ is a falling fuzzy implicative ideal of *A*.

**Proposition 45 .**

Let $\tilde{H}$ be a falling shadow of a random set *ξ* : *Ω* → *P*(*A*). If $\tilde{H}$ is a falling fuzzy implicative ideal of *A*, then we have, for any *x*, *y* ∈ *A*,

1. *Ω*((*x* ⊖ *y*) ⊖ *z*; *ξ*)∩*Ω*(*y* ⊖ *z*; *ξ*)⊆*Ω*(*x* ⊖ *z*; *ξ*);
2. *Ω*(*x*; *ξ*) = *Ω*(*x* ^*n*^; *ξ*), for any *n* ≥ 1;
3. *Ω*(*x*; *ξ*)∩*Ω*(*y*; *ξ*) = *Ω*(*x*∨*y*; *ξ*) = *Ω*(*x* ⊕ *y*; *ξ*);
4. *Ω*(*nx*; *ξ*) = *Ω*(*x*; *ξ*), for any *n* ≥ 1;
5. *Ω*(*x* ⊗ *y*; *ξ*)∩*Ω*(*x* ⊖ *y*; *ξ*) = *Ω*(*x*; *ξ*);
6. *Ω*((*x* ⊖ *y*) ⊖ *y*; *ξ*)⊆*Ω*(*x* ⊖ *y*; *ξ*).

**Proof**

1. For any *ω* ∈ *Ω*((*x* ⊖ *y*) ⊖ *z*; *ξ*)∩*Ω*(*y* ⊖ *z*; *ξ*), we have (*x* ⊖ *y*) ⊖ *z* ∈ *ξ*(*ω*) and *y* ⊖ *z* ∈ *ξ*(*ω*). Since *ξ*(*ω*) is an ideal of *A*, it follows that *x* ⊖ *z* ∈ *ξ*(*ω*); that is, *ω* ∈ *Ω*(*x* ⊖ *z*; *ξ*), and so *Ω*((*x* ⊖ *y*) ⊖ *z*; *ξ*)  *∩* *Ω*(*y* ⊖ *z*; *ξ*)  *⊆* *Ω*(*x* ⊖ *z*; *ξ*).
2. It is true for *n* = 1. For the case of *n* = 2, we have *Ω*(*x*; *ξ*) = *Ω*(1 ⊖ ¬*x*; *ξ*)  *⊇* *Ω*((1 ⊖ ¬*x*)⊖¬*x*; *ξ*)  *∩* *Ω*(¬*x* ⊖ ¬*x*; *ξ*) = *Ω*(*x* ^2^; *ξ*) by (1). On the other hand, since *x* ^2^ ≤ *x*, then *Ω*(*x*; *ξ*)⊆*Ω*(*x* ^2^; *ξ*) by Proposition 42 and Theorem 24, and so *Ω*(*x*; *ξ*) = *Ω*(*x* ^2^; *ξ*). Suppose that *n* ≥ 3 and *Ω*(*x*; *ξ*) = *Ω*(*x* ^*n*−1^; *ξ*), then we get *Ω*(*x*; *ξ*) = *Ω*(*x* ^*n*−1^; *ξ*)  =  *Ω*(*x* ^*n*−2^ ⊖ ¬*x*; *ξ*)  *⊇* *Ω*((*x* ^*n*−2^ ⊖ ¬*x*)  *⊖*  ¬*x*; *ξ*)  *∩* *Ω*(¬*x* ⊖ ¬*x*; *ξ*)  =  *Ω*(*x* ^*n*^; *ξ*). The other direction is that *Ω*(*x*; *ξ*)⊆*Ω*(*x* ^*n*^; *ξ*) since *x* ^*n*^ ≤ *x*, which shows that (2) is valid.
3. For any *x*, *y* ∈ *A*, we have *x*, *y* ≤ *x*∨*y* ≤ *x* ⊕ *y*, and so *Ω*(*x* ⊕ *y*; *ξ*)  *⊆* *Ω*(*x*∨*y*; *ξ*)  *⊆* *Ω*(*x*; *ξ*)∩*Ω*(*y*; *ξ*). On the other hand, since (*x* ⊕ *y*) ⊖ *x* = ¬*x*∧*y*, then *Ω*(*x* ⊕ *y*; *ξ*)  *⊇* *Ω*((*x* ⊕ *y*) ⊖ *x*; *ξ*)  *∩* *Ω*(*x*; *ξ*)  *⊇*  (*Ω*(¬*x*; *ξ*)  *∪* *Ω*(*y*; *ξ*))  *∩* *Ω*(*x*; *ξ*)  =  (*Ω*(¬*x*; *ξ*)∩*Ω*(*x*; *ξ*))  *∪*  (*Ω*(*y*; *ξ*)∩*Ω*(*x*; *ξ*))  =  *Ω*(1; *ξ*)  *∪*  (*Ω*(*y*; *ξ*)∩*Ω*(*x*; *ξ*))  =  *Ω*(*y*; *ξ*)∩*Ω*(*x*; *ξ*), and so it proves (3).
4. It is straightforward by (3).
5. On the one hand, *Ω*(*x*; *ξ*)  =  *Ω*(1 ⊖ ¬*x*; *ξ*)  *⊇* *Ω*((1 ⊖ (1 ⊖ *y*))⊖¬*x*; *ξ*)  *∩* *Ω*((1 ⊖ *y*)⊖¬*x*; *ξ*)  =  *Ω*(*x* ⊗ *y*; *ξ*)∩*Ω*(*x* ⊖ *y*; *ξ*). On the other hand, since *x* ⊗ *y* ≤ *x* and *x* ⊖ *y* ≤ *x*, we have *Ω*(*x*; *ξ*)⊆*Ω*(*x* ⊗ *y*; *ξ*)  *∩* *Ω*(*x* ⊖ *y*; *ξ*); that means (5) holds.
6. For any *ω* ∈ *Ω*((*x* ⊖ *y*) ⊖ *y*; *ξ*), we have (*x* ⊖ *y*) ⊖ *y* ∈ *ξ*(*ω*). Since *y* ⊖ *y* = 0 ∈ *ξ*(*ω*) and *ξ*(*ω*) is an implicative ideal of *A*, it follows that *x* ⊖ *y* ∈ *ξ*(*ω*), that is *ω* ∈ *Ω*(*x* ⊖ *y*; *ξ*), and thus *Ω*((*x* ⊖ *y*) ⊖ *y*; *ξ*)⊆*Ω*(*x* ⊖ *y*; *ξ*).

Some of equivalent conditions of falling fuzzy implicative ideals are given in the next theorem.

**Theorem 46 .**

Let $\tilde{H}$ be a falling shadow of a random set *ξ* : *Ω* → *P*(*A*). Then $\tilde{H}$ is a falling fuzzy implicative ideal of *A* if and only if for any *x*, *y*, *z* ∈ *A*,

1. *Ω*(*x*; *ξ*)⊆*Ω*(0; *ξ*);
2. *Ω*((*x* ⊖ *y*) ⊖ *z*; *ξ*)∩*Ω*(*y* ⊖ *z*; *ξ*)⊆*Ω*(*x* ⊖ *z*; *ξ*).

**Proof**

Assume that $\tilde{H}$ is a falling fuzzy implicative ideal of *A*, it follows that *Ω*(*x*; *ξ*)⊆*Ω*(0; *ξ*) by Lemma 23. (2) is directly from Proposition 45.

Conversely, assume that (1) and (2) hold. Let *ω* ∈ *Ω* and *x* ∈ *ξ*(*ω*). Then *ω* ∈ *Ω*(*x*; *ξ*)⊆*Ω*(0; *ξ*); that is, 0 ∈ *ξ*(*ω*). For any *x*, *y*, *z* ∈ *A*, if (*x* ⊖ *y*) ⊖ *z* ∈ *ξ*(*ω*) and *y* ⊖ *z* ∈ *ξ*(*ω*), then *ω* ∈ *Ω*((*x* ⊖ *y*) ⊖ *z*; *ξ*)∩*Ω*(*y* ⊖ *z*; *ξ*)⊆*Ω*(*x* ⊖ *z*; *ξ*), and so *x* ⊖ *z* ∈ *ξ*(*ω*). It follows that *ξ*(*ω*) is an implicative ideal of *A*. Thus $\tilde{H}$ is a falling fuzzy implicative ideal of *A*.

As a direct consequence of Theorem 46, we have the following result.

**Corollary 47 .**

Let $\tilde{H}$ be a falling shadow of a random set *ξ* : *Ω* → *P*(*A*). If $\tilde{H}$ satisfies the following conditions:

1. *Ω* = *Ω*(0; *ξ*);
2. *Ω*((*x* ⊖ *y*) ⊖ *z*; *ξ*)∩*Ω*(*y* ⊖ *z*; *ξ*)⊆*Ω*(*x* ⊖ *z*; *ξ*), for any *x*, *y* ∈ *A*,

then $\tilde{H}$ is a falling fuzzy implicative ideal of *A*.

The following result provides a condition for a falling shadow to be a falling fuzzy implicative ideal.

**Theorem 48 .**

Let *ξ* : *Ω* → *P*(*A*) be a random set and $\tilde{H}$ a falling shadow of *ξ*. Then $\tilde{H}$ is a falling fuzzy implicative ideal of *A* if and only if it satisfies

1. *Ω*(*x*; *ξ*)⊆*Ω*(0; *ξ*) for any *x* ∈ *A*;
2. *Ω*((*x* ⊖ (*y* ⊖ *x*)) ⊖ *z*; *ξ*)∩*Ω*(*z*; *ξ*)⊆*Ω*(*x*; *ξ*) for any *x*, *y*, *z* ∈ *A*.

**Proof**

Suppose that $\tilde{H}$ is a falling fuzzy implicative ideal of *A*. (1) is directly from Theorem 46. For any *ω* ∈ *Ω*((*x* ⊖ (*y* ⊖ *x*)) ⊖ *z*; *ξ*)∩*Ω*(*z*; *ξ*), we have (*x* ⊖ (*y* ⊖ *x*)) ⊖ *z* ∈ *ξ*(*ω*) and *z* ∈ *ξ*(*ω*); therefore *x* ⊖ (*y* ⊖ *x*) ∈ *ξ*(*ω*). Since (*y* ⊖ (*y* ⊖ *x*))⊖(*y* ⊖ *x*)⊖(*x* ⊖ (*y* ⊖ *x*))≤(*y* ⊖ (*y* ⊖ *x*)) ⊖ *x* = 0 ∈ *ξ*(*ω*), we obtain (*y* ⊖ (*y* ⊖ *x*))⊖(*y* ⊖ *x*) ∈ *ξ*(*ω*), and so *y* ⊖ (*y* ⊖ *x*) = *x* ⊖ (*x* ⊖ *y*) ∈ *ξ*(*ω*) by Proposition 45 and Lemma 5. Thus we have *ω* ∈ *Ω*(*x* ⊖ (*x* ⊖ *y*); *ξ*). Noticing that ((*x* ⊖ *y*) ⊖ *z*)⊖(*x* ⊖ (*y* ⊖ *x*)) = (*x* ⊖ (*x* ⊖ (*y* ⊖ *x*))) ⊖ *y* ⊖ *z* = (*x*∧(*y* ⊖ *x*)) ⊖ *y* ⊖ *z* = 0 ∈ *ξ*(*ω*), we get (*x* ⊖ *y*) ⊖ *z* ∈ *ξ*(*ω*). Since *z* ∈ *ξ*(*ω*), it follows that *x* ⊖ *y* ∈ *ξ*(*ω*); that is, *ω* ∈ *Ω*(*x* ⊖ *y*; *ξ*). Considering that *ξ*(*ω*) is an implicative ideal of *A*, we have *ω* ∈ *Ω*(*x* ⊖ *y*; *ξ*)∩*Ω*(*x* ⊖ (*x* ⊖ *y*); *ξ*)⊆*Ω*(*x*; *ξ*) by Theorem 27, and thus (2) is valid.

Conversely, for any *x*, *y*, *z* ∈ *A*, we get *Ω*(*x* ⊖ (*y* ⊖ *x*); *ξ*)⊆*Ω*(*x*; *ξ*) and *Ω*(*x* ⊖ *z*; *ξ*)∩*Ω*(*z*; *ξ*) = *Ω*((*x* ⊖ (*x* ⊖ *x*)) ⊖ *z*; *ξ*)∩*Ω*(*z*; *ξ*)⊆*Ω*(*x*; *ξ*), and so $\tilde{H}$ is a falling ideal of *A*. For any *ω* ∈ *Ω*((*x* ⊖ *y*) ⊖ *z*; *ξ*)∩*Ω*(*y* ⊖ *z*; *ξ*), we obtain (*x* ⊖ *y*) ⊖ *z* ∈ *ξ*(*ω*) and *y* ⊖ *z* ∈ *ξ*(*ω*). Since (*x* ⊖ *z*) ⊖ (*x* ⊖ (*x* ⊖ *z*)) ⊖ (*y* ⊖ *z*) ≤ (*x* ⊖ *y*) ⊖ (*x* ⊖ (*x* ⊖ *z*)) = (*x*∧(*x* ⊖ *z*)) ⊖ *y* ≤ (*x* ⊖ *z*) ⊖ *y* = (*x* ⊖ *y*) ⊖ *z*, and *ξ*(*ω*) is an ideal of *A*, then (*x* ⊖ *z*)⊖(*x* ⊖ (*x* ⊖ *z*)) ∈ *ξ*(*ω*); that is, *ω* ∈ *Ω*((*x* ⊖ *z*)⊖(*x* ⊖ (*x* ⊖ *z*)); *ξ*)⊆*Ω*(*x* ⊖ *z*; *ξ*). Thus *Ω*((*x* ⊖ *y*) ⊖ *z*; *ξ*)∩*Ω*(*y* ⊖ *z*; *ξ*)⊆*Ω*(*x* ⊖ *z*; *ξ*), it follows that $\tilde{H}$ is a falling fuzzy implicative ideal of *A*.

We can also give some conditions for a falling fuzzy ideal to be a falling fuzzy implicative ideal.

**Theorem 49 .**

Let $\tilde{H}$ be a falling shadow of a random set *ξ* : *Ω* → *P*(*A*). If $\tilde{H}$ is a falling fuzzy ideal of *A*, then the following conditions are equivalent:

1. $\tilde{H}$ is a falling fuzzy implicative ideal of *A*,
2. *Ω*((*y* ⊖ ¬*x*) ⊖ *z*; *ξ*)∩*Ω*(*x* ⊖ *y*; *ξ*)⊆*Ω*(*x* ⊖ *z*; *ξ*), for any *x*, *y*, *z* ∈ *A*;
3. *Ω*(0; *ξ*) = *Ω*((*x* ⊕ *x*) ⊖ *nx*; *ξ*) for any *x* ∈ *A* and *n* ≥ 1;
4. *Ω*(*x* ⊖ (*y* ⊖ *x*); *ξ*)⊆*Ω*(*x*; *ξ*), for any *x*, *y* ∈ *A*;
5. *Ω*((*x* ⊖ *y*) ⊖ *y*; *ξ*)⊆*Ω*(*x* ⊖ *y*; *ξ*), for any *x*, *y* ∈ *A*.

**Proof**

(1)⇔(2) Suppose that $\tilde{H}$ is a falling fuzzy implicative ideal of *A*. For any *x*, *y*, *z* ∈ *A*, we have *Ω*((*y* ⊖ ¬*x*) ⊖ *z*; *ξ*)∩*Ω*(*x* ⊖ *y*; *ξ*) = *Ω*((¬*z* ⊖ ¬*y*)⊖¬*x*; *ξ*)∩*Ω*(¬*y* ⊖ ¬*x*; *ξ*)⊆*Ω*(¬*z* ⊖ ¬*x*; *ξ*) = *Ω*(*x* ⊖ *z*; *ξ*), by Theorem 46.

Conversely, let *x*, *y*, *z* ∈ *A* be such that (*x* ⊖ *y*) ⊖ *z* ∈ *ξ*(*ω*) and *y* ⊖ *z* ∈ *ξ*(*ω*). It follows that *ω* ∈ *Ω*((*x* ⊖ *y*) ⊖ *z*; *ξ*)∩*Ω*(*y* ⊖ *z*; *ξ*) = *Ω*((¬*y* ⊖ *z*)⊖¬*x*; *ξ*)∩*Ω*(¬*z* ⊖ ¬*y*; *ξ*)⊆*Ω*(¬*z* ⊖ ¬*x*; *ξ*) = *Ω*(*x* ⊖ *z*; *ξ*), and so *x* ⊖ *z* ∈ *ξ*(*ω*). Noticing that *ξ*(*ω*) is an ideal of *A*, thus *ξ*(*ω*) is an implicative ideal, and therefore $\tilde{H}$ is a falling fuzzy implicative ideal of *A*.

(1)⇔(3) For any *ω* ∈ *Ω*(0; *ξ*), we have 0 ∈ *ξ*(*ω*). Since *x* ⊖ *x* = 0 ∈ *ξ*(*ω*) and ((*x* ⊕ *x*) ⊖ *x*) ⊖ *x* = (*x* ⊕ *x*)⊖(*x* ⊕ *x*) = 0 ∈ *ξ*(*ω*), taking into consideration that *ξ*(*ω*) is an implicative ideal, we have (*x* ⊕ *x*) ⊖ *x* ∈ *ξ*(*ω*); that is, *ω* ∈ *Ω*((*x* ⊕ *x*) ⊖ *x*; *ξ*), and thus *Ω*(0; *ξ*)⊆*Ω*((*x* ⊕ *x*) ⊖ *x*; *ξ*). Since (*x* ⊕ *x*) ⊖ *nx* ≤ (*x* ⊕ *x*) ⊖ *x*, then *Ω*(0; *ξ*)⊆*Ω*((*x* ⊕ *x*) ⊖ *x*; *ξ*)⊆*Ω*((*x* ⊕ *x*) ⊖ *nx*; *ξ*) by Theorem 24. It follows from Theorem 46 that *Ω*((*x* ⊕ *x*) ⊖ *nx*; *ξ*)⊆*Ω*(0; *ξ*); therefore, (2) holds.

Conversely, given *n* = 1, we get *Ω*(0; *ξ*) = *Ω*((*x* ⊕ *x*) ⊖ *x*; *ξ*) for any *x* ∈ *A*. It follows that (*x* ⊕ *x*) ⊖ *x* ∈ *ξ*(*ω*) for any *ω* ∈ *Ω*. Let *x*, *y*, *z* ∈ *A* be such that (*x* ⊖ *y*) ⊖ *z* ∈ *ξ*(*ω*) and *y* ⊖ *z* ∈ *ξ*(*ω*). By Lemma 5, we have (*x* ⊖ (*z* ⊕ *z*))⊖((*x* ⊖ *y*) ⊖ *z*)⊖(*y* ⊖ *z*) = ((*x* ⊖ *z*)⊖(*y* ⊖ *z*)) ⊖ *z* ⊖ ((*x* ⊖ *y*) ⊖ *z*)≤(*x* ⊖ *y*) ⊖ *z* ⊖ ((*x* ⊖ *y*) ⊖ *z*) = 0. Noticing that *ξ*(*ω*) is an ideal of *A*, we obtain *x* ⊖ (*z* ⊕ *z*) ∈ *ξ*(*ω*). Since (*z* ⊕ *z*) ⊖ *z* ∈ *ξ*(*ω*) and *x* ⊖ *z* ≤ ((*z* ⊕ *z*) ⊖ *z*)⊕(*x* ⊖ (*z* ⊕ *z*)), then *x* ⊖ *z* ∈ *ξ*(*ω*). Hence *ξ*(*ω*) is an implicative ideal, and thus $\tilde{H}$ is a falling fuzzy implicative ideal of *A*.

(1)⇒(4) It is immediate from the proof of Theorem 48.

(4)⇒(5) Since (*x* ⊖ *y*)⊖(*x* ⊖ (*x* ⊖ *y*)) = (*x* ⊖ *y*) ⊖ *y* for any *x*, *y* ∈ *A*, then *Ω*((*x* ⊖ *y*) ⊖ *y*; *ξ*)⊆*Ω*((*x* ⊖ *y*)⊖(*y* ⊖ (*x* ⊖ *y*)); *ξ*)⊆*Ω*(*x* ⊖ *y*; *ξ*), and thus (5) is valid.

(5)⇒(1) Noticing that (*x* ⊖ (*y* ⊖ *z*) ⊖ *z*) ⊖ *z* = (*x* ⊖ *z*)⊖(*y* ⊖ *z*) ⊖ *z* ≤ (*x* ⊖ *y*) ⊖ *z*, we have *Ω*((*x* ⊖ *y*) ⊖ *z*; *ξ*)⊆*Ω*((*x* ⊖ (*y* ⊖ *z*) ⊖ *z*) ⊖ *z*; *ξ*) = *Ω*(((*x* ⊖ *z*) ⊖ *z*)⊖(*y* ⊖ *z*); *ξ*). It follows that *Ω*((*x* ⊖ *y*) ⊖ *z*; *ξ*)∩*Ω*(*y* ⊖ *z*; *ξ*)⊆*Ω*(((*x* ⊖ *z*) ⊖ *z*)⊖(*y* ⊖ *z*); *ξ*)∩*Ω*(*y* ⊖ *z*; *ξ*)⊆*Ω*((*x* ⊖ *z*) ⊖ *z*; *ξ*)⊆*Ω*(*x* ⊖ *z*; *ξ*), and thus $\tilde{H}$ is a falling fuzzy implicative ideal of *A*.

**Proposition 50 .**

Let $\tilde{H}$ be a falling shadow of a random set *ξ* : *Ω* → *P*(*A*). If $\tilde{H}$ is a falling fuzzy implicative ideal of *A*, then one has

1. $\tilde{H}$ is a *T*_*m*_-fuzzy implicative ideal of *A*;
2. if *Ω*(*x*; *ξ*)⊆*Ω*(*y*; *ξ*) or *Ω*(*x*; *ξ*)⊆*Ω*(*y*; *ξ*) for any *x*, *y* ∈ *A*, then $\tilde{H}$ is a *T*_∧_-fuzzy implicative ideal of *A*;
3. for any *x*, *y* ∈ *A*, if *Ω*(*x*; *ξ*) and *Ω*(*y*; *ξ*) are independent random events, then $\tilde{H}$ is a *T*_*p*_-fuzzy implicative ideal of *A*.

**Proof**

The proof is similar to that of Theorem 30 and Proposition 31.

## Figures and Tables

**Figure 2:**